# A comprehensive mapping of stress system interactions with pain and their contribution to chronification of musculoskeletal pain: Protocol of the STRAIN study

**DOI:** 10.1371/journal.pone.0324089

**Published:** 2025-06-24

**Authors:** Matthijs Moerkerke, Joren Vyverman, Robrecht De Baere, Patricia Clement, Tom Smeets, Iris Coppieters, Inge Timmers, Jessica Van Oosterwijck

**Affiliations:** 1 Spine, Head and Pain Research Unit Ghent, Department of Rehabilitation Sciences, Faculty of Medicine and Health Sciences, Ghent University, Ghent, Belgium; 2 Pain in Motion International Research Consortium,; 3 Center of Research on Psychological disorders and Somatic diseases (CoRPS), Department of Medical and Clinical Psychology, Tilburg School of Social and Behavioral Sciences, Tilburg University, Tilburg, the Netherlands; 4 Pain in Motion Research Group (PAIN), Department of Physiotherapy, Faculty of Physical Education and Physiotherapy, Vrije Universiteit Brussel, Brussels, Belgium; 5 Department of Medical Imaging, Ghent University Hospital, Ghent, Belgium; 6 Department of Diagnostic Sciences, Faculty of Medicine and Health Sciences, Ghent University, Ghent, Belgium; PLOS: Public Library of Science, UNITED KINGDOM OF GREAT BRITAIN AND NORTHERN IRELAND

## Abstract

**Background:**

Stress is suggested to be an important factor contributing to the development and persistence of musculoskeletal pain. Although stress and pain interactions are well known, it remains largely unclear how (dys)function of the major stress systems (i.e., the autonomic nervous system and the hypothalamic-pituitary-adrenal axis) contributes to pain extent and duration, and what the underlying mechanisms are. A comprehensive characterization of the stress systems and their interactions with pain is needed to better understand how stress confers vulnerability for persistent pain.

**Aims:**

The primary aim of this study is to characterize stress system (dys)functioning (i.e., including basal levels, reactivity and recovery to acute stress, and chronic stress levels) in musculoskeletal pain groups with varying pain duration and extent, and to investigate the interaction between stress and pain at the psychosocial, (psycho)physiological and neural level. The secondary aim is to define the contribution of stress to pain trajectories, including chronification and recovery.

**Methods:**

A study with a cross-sectional and a longitudinal arm will be conducted in musculoskeletal pain groups with varying pain characteristics, including chronic widespread pain (fibromyalgia), chronic and subacute localized pain (low back pain), and pain-free controls (n = 35/group). To characterize pain trajectories (recovery/persisting), localized pain groups will be reassessed after six months. Stress and pain characteristics and functionality will be assessed using questionnaires, autonomic measures (alpha-amylase, blood pressure, heart rate, respiratory rate, skin conductance and skin temperature), hormonal concentrations (cortisol and oxytocin), quantitative sensory testing (pain thresholds, pain tolerances, conditioned pain modulation and temporal summation of pain), and magnetic resonance imaging (brain structure and function).

**Discussion:**

This study will provide crucial insights in the role of stress in the extent of pain symptomatology and in conferring risk for pain chronicity. Additionally, it will shed light on the underlying mechanisms of stress and pain interactions.

Trial registration: ClinicalTrials.gov (NCT06892977)

## 1. Introduction

Stress and pain are intricately and dynamically connected at various levels, both in acute and chronic contexts. Acute stress can influence pain perception, either amplifying or reducing sensitivity to painful stimuli in individuals with and without clinical pain [1–5], and can magnify self-perceived pain in individuals with chronic pain [6]. Conversely, pain can function as a stressor by triggering activation of the major stress response systems, i.e., the autonomic nervous system (ANS) and the hypothalamic-pituitary-adrenal (HPA) axis. The sympathetic branch of the ANS, also called the sympatho-adreno-medullary (SAM) system, increases the release of catecholamines (e.g., noradrenaline) in response to a stressor, leading to physiological changes including elevations in heart rate and blood pressure. This sympathetic activation suppresses parasympathetic activity, which in turn elicits bodily changes such as an increase in heart rate and a reduction in heart rate variability. Besides these changes in electrophysiological parameters, stress can swiftly elevate enzyme concentrations in saliva such as alpha-amylase (sAA) [7]. The SAM system is characterized by its rapid activation and quick recovery during and following a stressor. In contrast, the HPA axis has a delayed but longer lasting response through the release of cortisol, a well-known stress hormone that influences energy metabolism, cardiovascular function and the immune system [8,9]. Following a stressor, HPA axis activity is regulated by a negative feedback system that is at least in part mediated by limbic and frontal brain structures (e.g., amygdala, hippocampus, medial prefrontal cortex), to promote recovery and restore homeostasis [10]. Cortisol plays an important part in this recovery, although there are also other hormones involved in regulating the HPA axis’ activity, including oxytocin, a neuropeptide traditionally associated with social bonding and childbirth [11]. Given that our body’s response to stress is orchestrated within multiple systems and time scales, a comprehensive assessment of stress is warranted to gain a full picture of its interactions with pain.

While both acute stress and acute pain are designed to promote survival, persistence of either stress or pain is maladaptive and negatively affects well-being. Research has shown support for both elevated stress levels and dysregulation of the stress systems in people with persistent pain [12]. People suffering from chronic musculoskeletal (MSK) pain generally report higher levels of emotional distress compared to pain-free individuals [13]. Additionally, alterations in ANS functioning have been observed in chronic MSK pain conditions, as indicated by increased sympathetic and decreased parasympathetic modulation [14], blunted ANS reactivity (e.g., during a cognitive stressor) [15,16], and diminished or delayed parasympathetic reactivation during recovery from an acute stressor [15]. When stress is sustained over time, the HPA axis can become dysregulated, manifesting as either overactivity or underactivity of the system [17] . Signs of HPA axis dysregulation are observed in chronic MSK pain too, as both heightened and reduced basal cortisol levels, as well as increased and diminished cortisol reactivity [12] have been observed. These inconsistent findings might be explained not only by limited assessments of stress, but also by variations in duration and extent of the pain symptoms. Since pain is a potent stressor, individuals suffering from MSK pain may initially experience higher cortisol levels, which may lead to a rapid depletion of cortisol in response to repeated or persistent stress, potentially resulting in reduced cortisol levels as pain duration increases. This hypothesis is supported by observations in people with fibromyalgia, a widespread MSK pain condition, in which patients with a shorter disease duration showed increased hair cortisol levels, as a marker of chronic stress, while those with long disease duration show lower levels of hair cortisol (indicating a depletion of cortisol levels) [18]. This shows the importance of not only measuring hormones over a short (acute) period of time using saliva, serum, or urine samples, but to also include markers of longer (chronic) periods of cortisol exposure (e.g., cortisol concentrations from hair samples [19]). Yet, whether and how the multiple stress systems contribute to differences in pain extent and duration, and whether teasing those apart may explain some of the reported inconsistencies, remains to be tested.

A recent systematic review and meta-analyses from our group has shown evidence for the association of pain sensitivity with baroreceptor dysregulation and HPA axis dysfunction [20]. Findings showed that when acute stress is induced, an association between autonomic dysfunction and higher pain sensitivity can be observed in patients with chronic pain. However, the review also illustrates that studies investigating the association between HPA axis functioning and experimental pain are limited. More research investigating the interactions between both the ANS and the HPA axis with static and dynamic experimental pain paradigms, and exploring the reactivity and recovery of these stress systems with standardized, effective stressors is necessary to pinpoint underlying mechanisms of the pain-stress interactions [20]. On a neural level, research has shown that when pain becomes chronic, brain activity involved in sensory processing shifts towards brain networks engaged in emotional processing (i.e., the corticolimbic system [21]. Interestingly, these networks overlap with areas involved in stress response regulation [21], suggesting shared mechanisms. A study reported that individuals with chronic MSK pain as well as pain-free controls with stronger cortisol responses to an acute painful stressor showed reduced pain-related activation in the insula and somatosensory cortex [22], thus illustrating the stress-pain interaction on a neural level too. While there is clear evidence linking pain with stress at multiple levels, how stress system dysfunction contributes to increased risk for pain persistence, and what the underlying (psycho)physiological and neural mechanisms are largely unclear.

To shed light on the underlying mechanisms of pain and stress interactions and their contribution to pain extent and chronification, the aim of the current STRAIN (‘stress in pain’) study is to characterize stress system functioning and its relation to pain in individuals with subacute versus chronic, and localized versus widespread MSK pain, to allow comparison of groups with different pain characteristics in terms of pain extent and duration. A comprehensive mapping of both ANS and HPA axis stress systems will be used to assess basal levels, reactivity and recovery of acute stress, and chronic stress exposure. Interactions between stress and psychosocial, (psycho)physiological, and neural correlates of pain will be investigated. Additionally, the secondary aim of this study is to characterize stress system responses over time and their role in MSK pain recovery and chronification. Therefore, stress system functioning and its interactions with pain will be reexamined after six months in the patients with subacute MSK pain.

## 2. Materials and methods

### 2.1. Study design

The STRAIN study protocol was pre-registered at ClinicalTrials.gov (NCT06892977) and reported in accordance with the Strengthening the Reporting of Observational Studies in Epidemiology (STROBE) statement [23]. Recruitment for this study commenced on 8 February 2025 and is anticipated to conclude by 1 June 2026. Study completion and the release of initial results are expected by 1 December 2026. The study consists of a cross-sectional arm and a longitudinal arm (i.e., follow up at six months). A total of 140 participants will be recruited for this study, across four groups (n = 35/group): (1) a group with chronic widespread MSK pain (i.e., fibromyalgia), (2) a group with chronic localized MSK pain (i.e., non-specific low back pain (LBP) for ≥six months), (3) a group with subacute localized MSK pain (i.e., non-specific LBP for <three months), and (4) a pain-free control group (no history of chronic pain or current pain). All groups will participate in the cross-sectional arm which consists of a four-hour lab assessment at Ghent University (Ghent, Belgium) and three (consecutive) days of home assessments. Participants of the localized MSK pain groups will also participate in the longitudinal arm consisting of the same assessments, supplemented with assessing pain persistence or recovery. The study will be performed in accordance with the Code of Ethics of the World Medical Association (Declaration of Helsinki) and the international ethical and scientific quality standards accepted by the International Conference on Harmonization guidelines for Good Clinical Practice. Several patient-groups were involved in various stages of developing the study protocol, providing insights into the research and helping to minimize participant burden. The study protocol and data management plan were approved by the Medical Ethics Committee of Ghent University/Ghent University Hospital and all participants will provide written informed consent prior to study participation.

### 2.2. Participants

Participants will be recruited through an existing database of volunteers for pain research, advertisements (e.g., social media, newsletters), and the distribution of flyers at private practices, (university) hospitals and public places.

All participants must be Dutch speaking, aged between 18 and 45 years, and have a body mass index between 18.5 kg/m^2^ and 35 kg/m^2^. Inclusion criteria for patients in the MSK pain groups are an average pain intensity of ≥2/10 on a visual analogue scale (VAS) and a pain-related disability of ≥14/70 on the Pain Disability Index (PDI) [24]. Participants of the chronic widespread MSK pain group will require proof of a primary fibromyalgia with chronic widespread pain diagnosis. As different diagnostic criteria have been used over time, researchers will check whether participants comply with the 2010 ACR-criteria [25]. To ensure that pain complaints are of MSK nature, only individuals with non-specific (primary) LBP will be included, with those suffering from LBP less than three months being eligible to participate in the subacute localized MSK pain group and those with LBP equal or more than six months being eligible to participate in the chronic localized MSK pain group. Pain-free controls should not report current pain, a history of chronic pain, or treatment for a pain condition in the last six months.

Anyone using hormonal replacement therapy, drugs ≥ 1x/week, having no stable use of medication (if applicable) one month before the study visit, a contra-indication for magnetic resonance imaging (MRI; i.e., electrical or mechanical implants, electrodes, or metal fragments in the body, being claustrophobic, weighing >150 kg), or being menopausal, pregnant, less than one year postnatal or lactating will be excluded. Furthermore, having a (history of a) psychiatric, cardiovascular, endocrine, or neurological condition (e.g., diabetes, epilepsy), spinal traumata, spinal surgery, or a severe communicative disorder or cognitive disability will not be eligible. The Dutch version of the simplified version of the Mini International Neuropsychiatric Interview (MINI) [26] based on the Diagnostic and Statistical Manual of Mental Disorders, Fifth edition (DSM-V) will be consulted in case of the presence of potential psychiatric conditions. People with LBP will be excluded if they suffer from specific LBP (i.e., LBP due to a clear identifiable pathology, e.g., lumbar radiculopathy).

Demographical characteristics and potential confounders will be assessed, including age, biological sex assigned at birth and gendered pain dynamics, height and weight (body mass index), ethnicity, hormonal cycle and contraceptives, medication use, drugs use, alcohol use, nicotine use, educational level, occupation, comorbidities, sleep experience (quality and quantity), and used pain management strategies. These will also be used to examine whether participants complied with the instructions described below.

### 2.3. Setting

Eligibility will be assessed through an online screening questionnaire and potential participants will be contacted for additional information, if required. Eligible participants will receive a secure link to health-related questionnaires ten days prior to the study. Research Electronic Data Capture (REDCap) software [27,28], a secure web-based software platform designed to facilitate data collection and management for research, will be used to collect self-reported data online.

Participants will be asked to adhere with following instructions in preparation for the lab assessments (if applicable and medically permissible): be awake for ≥two hours, refrain from consuming nicotine, other beverages than (still) water, and food two hours prior to the study visit [4], strenuous mental or physical activity <24 hours prior to the study visit [29], opioid use < 24 hours prior to the study visit [30], postpone the intake of medication with analgetic effects or affecting blood pressure until after the study visit [31,32], and maintain a normal sleeping routine before and three days after the study visit. A normal sleep routine is defined as going to bed and waking up at the time that is most common in the participant’s daily routine.

**Fig 1:**
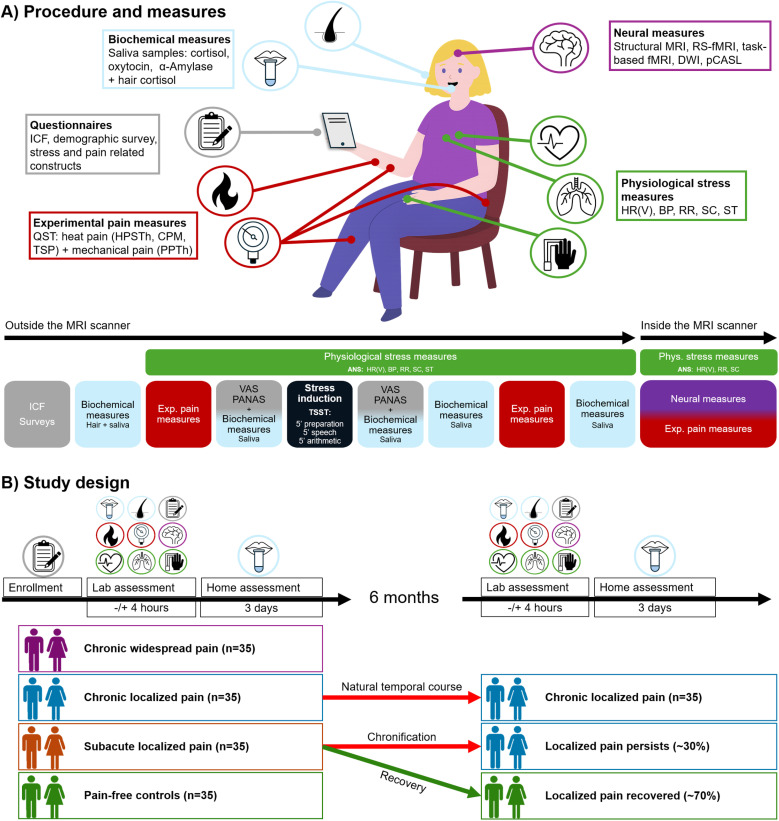
Procedure, measures and study design. **A.** Procedure and measures. Questionnaires include the informed consent form (ICF), a demographic survey and stress and pain related constructs (i.e., questionnaires). Biochemical measures include five saliva samples during the lab assessment to measure cortisol, oxytocin and alfa-amylase and include one hair cortisol sample. Experimental pain measures include CPM (conditioned pain modulation), TSP (temporal summation of pain), HPSTh (heat pain suprathreshold) and PPTh (pressure pain threshold). Physiological stress measures include HR(V) (heart rate (variability)), BP (blood pressure), RR (respiratory rate), SC (skin conductance) and ST (skin temperature). Neural measures include structural MRI, RS-fMRI (resting-state fMRI), task-based fMRI (experimental pain – functional MRI), DWI (diffusion-weighted MRI) and pCASL (Pseudo-Continuous Arterial Spin Labeling). Before and after stress induction, using the TSST (Trier Social Stress Test), VAS (Visual Analogue Scale) and PANAS (Positive And Negative Affect Schedule) are acquired. **B.** Study design. After enrollment and the lab assessment (as displayed in A), participants collect five saliva samples at home throughout the day for three consecutive days. The chronic and subacute low back pain groups are tested again, using the same test-battery, after a follow-up period of six months.

The lab assessment consists of (1) assessments of stress (using self-reported and physiological measures), (2) assessments of pain (using self-reports, quantitative sensory testing (QST)), (3) assessments of the brain (using (functional)MRI). After the study visit, participants will be instructed to collect saliva samples at home for three days as part of the physiological stress assessments. After a follow-up period of six months, the same assessments will be repeated. In addition, pain persistence and recovery will be assessed using a questionnaire. An overview of the procedures, measures and study design is depicted in Fig 1. For an overview of all measures and their timing, see Table S1.

### 2.4. Assessments of stress

Both non-induced (basal) and experimentally induced stress responses, and both acute (via self-reports, ANS measures and salivary hormonal levels) and chronic (via hair cortisol) states will be measured.

#### 2.4.1 Self-reported stress.

An overview of the stress-related trait questionnaires and timing of administration can be found in Table 1.

**Table 1:** Overview of the validated stress- and pain-related questionnaires.

Questionnaire	Domain	Timeline		
		Screening	Baseline	6-month follow-up
**Hospital Anxiety and Depression Scale** [33]	Anxiety and depression			
**Pain Anxiety Symptoms Scale-20** [34]	Fear and anxiety specific to pain			
**Pain Catastrophizing Scale** [35]	Pain catastrophizing			
**Pain Disability Index** [36]	Daily functioning related to pain			
**Pain Vigilance and Awareness Questionnaire** [37]	Attention to pain			
**Brief Resilience Scale** [38]	Recovery or bouncing back from stress			
**Intolerance Uncertainty Scale-12** [39]	Responses to uncertainty, ambiguous situations and the future			
**Perceived Stress Scale** [40]	Perception of stress			
**Stress Mindset Measure** [41]	Perception of stress as enhancing or debilitating			
(Shortened) **Pittsburgh Sleep Quality Index** [42]	Sleep quality			
**Childhood Trauma Questionnaire** [43]	Childhood trauma			

A VAS will be used to assess the effects of the acute stress induction on self-perceived stress (see Fig 1). The VAS will range from zero to 100 mm, with zero representing “no stress at all”, and 100 representing “most stress imaginable” [44]. The change in self-perceived stress will be calculated by subtracting VAS-scores (Δ self-perceived stress = post-stressor VAS-scores – pre-stressor VAS-scores).

Positive and negative affect will be assessed using the Positive and Negative Affect Schedule (PANAS) [45]. The change in positive and negative affect due to a stressor will be calculated by subtracting PANAS values (Δ positive and negative affect = post-stressor values – pre-stressor values) [4]. The PANAS consists of ten questions concerning positive affect, and ten questions concerning negative affect. Each question is scored between one and five, with one being “very slightly or not at all”, and five being “extremely”. For both categories, the final score ranges between ten and 50, with higher scores representing higher levels of positive or negative affect.

Participants will fill out the Primary Appraisal Secondary Appraisal (PASA) to examine their perception of an upcoming stressor, either as threatening or challenging, using a Likert scale [46].

#### 2.4.2 ANS measures – acute autonomous stress markers.

Saliva will be collected using a synthetic Salivette (Sarstedt, the Netherlands) at several time points across the lab assessment (see also 4.4). Participants will be instructed to place the synthetic swab in their mouths under the tongue for two minutes until it becomes saturated, without chewing on it. After collection, saliva samples will be immediately stored in a freezer (−20°C) and will be registered at the UZ Ghent Biobank. sAA concentrations (nmol/l) will be determined from the collected saliva samples using enzyme-linked immunosorbent assay (ELISA) (Dresden LabService GmbH).

Continuous physiological monitoring will be performed during the entire lab assessment using the wireless and portable telemetry data acquisition system NeXus-10 MkI (Mind Media BV) with BioTrace+ software (Mind Media BV) and an MRI-compatible BIOPAC data acquisition system (BIOPAC Systems Inc) with AcqKnowledge software (BIOPAC Systems Inc). Respiratory rate (RR) will be measured using a piezoelectric stretch sensor placed around the chest and is expressed in the number of breaths per minute. Electrocardiography (ECG) will be used to measure heart rate (HR). HR will be expressed in beats per minute (bpm); HR variability (HRV) as the root mean square of successive differences between the beat-to-beat or NN intervals (RMSSD), and as the low (LF; 0.04–0.15 Hz) and high frequency (HF; 0.15–0.40 Hz) power components calculated from frequency analyses following fast Fourier transformation. The vertical lead II electrode placement will be used, which is in accordance with BioTrace+ for NeXus-10 software’s recommendations (Mind Media BV). In the MRI environment, the electrodes will be positioned closer together to minimize artifacts in the ECG trace [47]. Skin conductance (SC) will be measured using two electrodes placed on the non-dominant hand and expressed in micro siemens (µS). Skin temperature (ST) will only be measured in the lab (i.e., outside the MRI scanner) using one electrode placed on the non-dominant palmar side of the distal phalanx of the third digit and expressed in degree Celsius (°C). Systolic and diastolic blood pressure (BP) (Omron X3 Comfort) will be measured intermittently by using an inflatable cuff placed around the non-dominant upper arm, which will be supported and positioned at heart level. BP is expressed in millimeters of mercury (mmHg).

To establish a baseline for physiological monitoring, participants will focus on a fixation cross to minimize distractions. A similar fixation cross will be used during MRI scans, specifically between the pain stimuli. Before and after the acute stress induction in the lab, participants will be instructed to look at a neutral five-minute video to ensure consistency (e.g., in emotional arousal) in physiological monitoring across participants.

### 2.5 Hormonal stress markers

#### 2.5.1 HPA axis – acute stress hormone levels.

Cortisol and oxytocin concentrations (pg/µl) will be determined from the collected saliva samples using the same procedures as mentioned for salivary alpha-amylase in 4.2. These hormones and alpha-amylase will be analyzed with liquid chromatography with coupled tandem mass spectrometry (LC-MS/MS) method (Dresden LabService GmbH).

#### 2.5.2 HPA axis – chronic stress hormone levels.

Long-term stress, over the last three months, will be assessed by means of hair cortisol concentrations (pg/mg). One sample will be taken at the start of the experiment by cutting hair from the posterior vertex region (i.e., ± 0.5 cm^2^, three centimeters (cm) in length covers three months). Samples will be collected in aluminum foil, stored in a dry and dark place, and analyzed by the LC-MS/MS method (Dresden LabService GmbH).

### 2.6 Timing and conditions of stress level assessments.

Stress-related questionnaires will be acquired ten days before and throughout the experiment. Induced stress responses will be assessed in the lab (i.e., outside the MRI). VAS scores for stress will be administered before and after the acute stress induction. The PANAS will be administered at baseline, before and after the acute stress induction and the PASA will be administered before the acute stressor induction. Saliva samples will be collected at arrival (t1), prior to (t2), immediately after (t3), 15 minutes after (t4) and 50 minutes after (t5) the end of acute stress induction. RR, ECG, SC and ST assessments prior to and following the stress induction will be taken over five-minute windows [48,49]. BP will be measured at baseline, before stress induction, immediately after stress induction and five, ten and fifteen minutes after stress induction.

Acute stress will be induced using the Trier Social Stress Test (TSST), which is considered a gold standard to induce psychosocial stress without using a physical stressor (pain) [50]. The TSST consists of a brief instruction, a five-minute preparation phase, a five-minute social speaking task and a five-minute mental arithmetic task. The task includes negative feedback from a jury and a camera positioned in front of the participant. Participants will be instructed to look directly at the jury or the camera. They will be falsely informed that their behavior and facial expressions will be recorded and analyzed afterwards. Since habituation of the TSST might occur for the reassessment (at six-month follow-up), different social speaking and mental arithmetic tasks will be employed (e.g., job interview, ideal mentor; subtracting 17 from 2043 or from 1431) [51].

Non-induced stress system functioning will be assessed at home. Participants will collect saliva samples for three (consecutive) days. Samples at home will be acquired directly after awakening (timepoint at home one or h1), 30 minutes after awakening (h2), 45 minutes after awakening (h3), at 16.00h (h4) and at 21.00h (h5). This will allow characterization of the first sample (h1), the cortisol awakening response (CAR) (mean increase = (h2 + h3/ 2*h1)), and basal cortisol levels throughout the day (Area Under the Curve (AUC) h1-h5) [52]. Self-collection has been previously used in chronic MSK pain [53]. Adherence to the instructions will be assessed, and the timing of sampling will be verified through documentation of the timepoint (by taking a picture of the sample with a timestamp) at which each sample is collected.

### 2.7. Assessments of pain

Different aspects of pain will be examined using self-reports and experimental pain paradigms (i.e., QST) to elicit and assess acute pain responses before and after acute stress, and during fMRI.

#### 2.7.1. Self-reported pain.

An overview of the pain-related trait questionnaires and timing of administration can be found in Table 1. A VAS involving a slider will be used to assess self-perceived pain intensity effects at baseline, and before and after the acute stress induction (i.e., pain intensity and pain unpleasantness). A range from zero to 100 will be used, with zero representing “no pain/unpleasantness at all”, and 100 representing “most pain/unpleasantness imaginable” [44]. The change in self-perceived pain intensity/ unpleasantness will be calculated by subtracting VAS-scores (Δ self-perceived pain = post-stressor VAS-scores – pre-stressor VAS-scores).

Pain origin, pain onset, last pain, pain evolution, pain pattern, pain throughout the day, pain quality, pain intensity, and pain location will be reported in the ten days prior to the start of the experiment or in the screening. Current pain intensity and pain location will be questioned on the day of the experiment itself.

#### 2.7.2. Quantitative Sensory Testing.

A static and dynamic QST battery will be used to assess pressure pain thresholds (PPTh), heat pain threshold (HPTh), heat pain tolerance (HPTo), conditioned pain modulation (CPM), and tonic temporal summation of pain (TSP).

To determine the presence of local and/or widespread hyperalgesia, PPTh will be assessed using a digital algometer with a one cm rubber tip (FDX50, Wagner Instruments, USA, Range 250 x 0.2N) on the following bilateral body sites locations: the proximal one-third of the calf, on the gastrocnemius muscle [54,55], five cm lateral to the third lumbar vertebra at the level of the lumbar vertebral column on the erector spinae muscle [54–56], and five cm distal to the lateral epicondyle of the humerus on the extensor carpi radialis muscle [54,57]. Each assessment will start at the left side, and the order of locations will be randomized. Participants will sit with 90° flexion in the knees, a neutral to slightly flexed low back position, and the forearms in a prone position with slight elbow flexion (30°), supported by a table. The pressure will be build up at one kilogram per second, applied perpendicular to the muscle fiber direction, and participants will verbally indicate when they first experience discomfort [58]. The PPTh measurement will be performed three times at each location, with a ten-second interval between consecutive measurements to prevent skin sensitization [59,60]. The first measurement will be used for familiarization and discarded from analysis. The average from the second and third measurement will be used to calculate the mean PPTh [58].

Pain sensitivity of heat stimuli will be evaluated using the TSA-2 with a three cm contact probe (Medoc Ltd.). Utilizing the principles of the Peltier element, the passage of a current through the device induces temperature changes, which are controlled by an active feedback system. Once the target temperature is reached, the temperature will be actively adjusted back to the baseline temperature (i.e., 35°C). The contact probe will be placed three cm distally from the elbow in the center of the volar part of the non-dominant forearm, and participants will be provided with a dual-button response device. The thermal stimulus will start at baseline temperature and will increase at a controlled rate of 0.3°C/s. The participants will be instructed to press the blue button when the temperature becomes unpleasant (HPTh), and to press the red button when the temperature becomes intolerable (HPTo). The HPTh and HPTo will be determined three times with an interstimulus interval of 30 seconds to avoid skin sensitization. The heat pain suprathreshold (HPSTh, which is the average of HPTh values and HPTo values [61]) will be used as stimulus intensity in the CPM and TSP test paradigms.

To assess central pain facilitation, a tonic TSP test paradigm will be executed. The three cm thermode (Medoc Ltd.) will be placed three cm distally from the elbow in the center of the volar part of the dominant forearm and will be secured to the testing site with a velcro strap. Temperature will rise from baseline and will reach HPSTh temperature in 1.25 seconds. Therefore, the rate of temperature increase will vary depending on the temperature corresponding to the HPSTh. The heat stimuli will be applied for 117.5 seconds, after which the temperature will reach baseline again in 1.25 seconds, resulting in a total duration of 120 seconds [61]. Pain intensity will be assessed at 1.25 (T1.25 sec), 5, 10, 20, 40, 60, 80, 100 and 118.75 (T118.75 sec) seconds on a verbal rating scale ranging from zero to 100 (zero = “no pain”; 100 = “most pain imaginable”) after showing a VAS [44].

To assess the dysfunction of descending pain inhibitory pathways, a parallel CPM paradigm will be conducted. Two three-by-three cm thermodes (Medoc Ltd.) will be positioned three cm distally from the elbow in the center of the volar part of the forearm, with one thermode (corresponding to the test stimulus) applied to the dominant side, and the other to the non-dominant side (corresponding to the conditioning stimulus). First, heat stimuli will be elicited on the dominant forearm. Starting from baseline, the HPSTh temperature will be reached in 1.25 seconds and will last for 7.5 seconds. This is the test stimulus (TS1). After an interstimulus interval of 45 seconds, temperature will increase on the non-dominant forearm, will reach the HPSTh temperature after 1.25 seconds, and will maintain for 22.5 seconds. This is the conditioning stimulus (CS). Fifteen seconds after the start of the CS, the TS will be applied again on the dominant forearm (TS2) [62]. Pain intensity for TS1 and TS2 will be assessed just before the temperature starts declining (at 8.75 sec) and for CS 15 seconds after application, using a verbal rating scale ranging from zero to 100 (zero = “no pain”; 100 = “most pain imaginable”) after showing a VAS [44]. The magnitude of CPM will be determined by subtracting the verbal rating score of the TS in the presence of the CS from the verbal rating score of the TS alone (Δ CPM = TS_2_ - TS_1_). A negative change score reflects an anti-nociceptive response, indicating effective CPM, whereas a positive change score suggests pronociceptive response and inefficient CPM [60].

### 2.8. MRI of the brain

**Fig 2:**
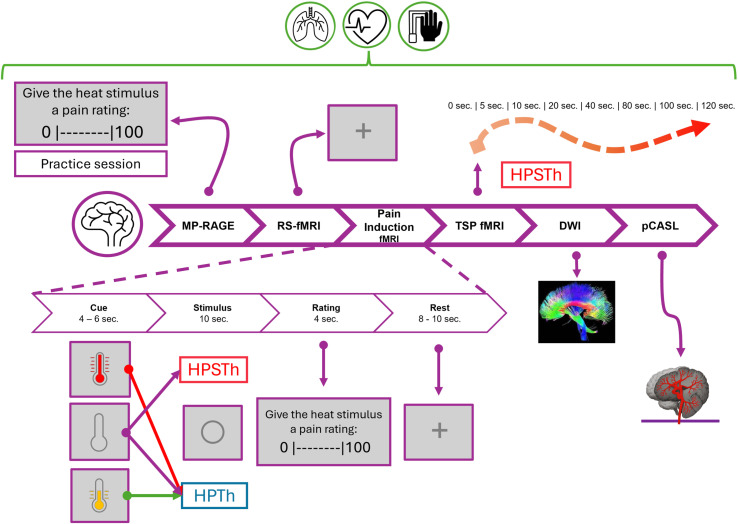
Overview MRI scanning procedure. Neural measures include MP-RAGE (Magnetization Prepared-RApid Gradient Echo) anatomical data, RS-fMRI (Resting-State fMRI), two task-based fMRI acquisitions (pain induction and TSP [Temporal Summation of Pain]), HPSTh (heat pain suprathreshold), HPTh (heat pain threshold), DWI (Diffusion-Weighted Imaging) and pCASL (Pseudo-Continuous Arterial Spin Labeling).

An overview of the MRI scanning procedure can be found in Fig 2. Before entering the MRI room, an MRI safety checklist will be filled out by the participant, and the researcher will check the presence of any contra-indications and the removal of metal objects. Images of the brain will be acquired with a three Tesla MAGNETOM Prisma Fit using a 64-channel head coil (Siemens, Erlangen, Germany) MRI Scanner at the Core Ghent Institute for functional and Metabolic Imaging (GIfMI) at Ghent University, Belgium. As part of standard practice, images are checked visually during scanning and scans will be repeated if necessary (e.g., due to movement or other artefacts). Visual stimuli and instructions will be presented to the participant on an MRI-proof 32 UHD BOLD-screen through a Python script, using PsychoPy packages [63]. The following scanning sequence will be used to evaluate brain structure and function: (1) localizers, (2) T1-weighted structural MRI, (3) Resting-State functional MRI (RS-fMRI), (4) task-based fMRI, (5) field map, (6) Diffusion-Weighted Imaging (DWI), and (7) multi-post labeling delay (PLD) pseudo-Continuous Arterial pin Labeling (pCASL). The set of localizer scans will be used to determine the correct orientation and positioning for the subsequent scans.

T1-weighted anatomical data will be obtained through Magnetization Prepared-RApid Gradient Echo (MP-RAGE), with a repetition time (TR) of 2250 milliseconds (ms), echo time (TE) of 4.18 ms and a slice thickness of 1.00 millimeters (mm) to determine macro-structural properties of the gray matter (i.e., surface area, cortical thickness and volume). During this scan, participants will be able to practice using the fMRI trackball system (NAtA Technologies), which will be used for providing ratings during the task-based functional scans.

Both RS and task-based fMRI scans will be acquired through echo planar imaging with a multiband factor of 4, with a TR of 1070 ms, a TE of 31 ms and a slice thickness of 2.5 mm. 60 slices of 2.5 mm will be acquired with a flip angle of 52 degrees. This set of functional imaging will be followed by a field map to facilitate possible artifact corrections during postprocessing.

RS-fMRI scans will be performed to evaluate functional connectivity relating to pain processing. Participants will be instructed to focus on a fixation cross and to let the mind wander.

During the first task-based fMRI scans acute brain responses to painful heat stimuli and their anticipation will be captured. Each trial will first consist of an anticipation phase between four and six seconds, during which a cue is presented that signals either a more painful (red thermometer), less painful (orange thermometer), or unknown (neutral; empty thermometer) stimulus. Participants are instructed on the meaning of the cues beforehand. The cue will be followed by a heat stimulation period of ten seconds, at an intensity equal to either the HPTh or the HPSTh, which will be recalibrated when the participant takes place in the scanner. To elicit heat stimuli, a three-by-three cm MRI-proof thermode (Medoc Ltd.) will be placed through a waveguide and will be applied on the volar part of the forearm. The combination between cue and stimulation intensity is randomized between trials and four conditions will be tested: (A) neutral cue + HPTh; (B) neutral cue + HPSTh; (C) more-pain cue + HPTh and; (D) less-pain + HPTh. Conditions (A) and (B) will allow for investigating the brain’s response to heat pain, while conditions (C) and (D) will allow for investigating the effect of cues (anticipation) on pain processing. Each condition will be repeated six times, and three additional trials (more-pain cue + HPSTh) will be added to reinforce the suggestion that the more-pain cue is associated with a more painful stimulus, resulting in a total of 27 trials. During heat pain stimulation, participants are told to focus on a central shape presented in the center of the BOLD-screen, reminiscent of the cues presented just before to reduce the level of visual difference. After each stimulation phase, the participant is allotted four seconds to recall and rate the pain intensity between zero and 100 via the fMRI trackball system by scrolling through the slider component. Then, a fixation cross is presented for eight to ten seconds (jittered), allowing participants to rest before the next trial starts.

During the second task-based MRI scans, TSP will be evaluated in the same way as outside the MRI scanner as described in 5.2. However, the stimulus (of two minutes) will be repeated three times, with an interstimulus interval of 30 seconds, to prevent skin sensitization. The participant will once again fixate on the same shape as during the previously described stimuli, and on the fixation cross during the baseline phases. The method for scoring pain intensity is identical as during the previous task.

DWI scans will be acquired using multiband with a factor of three [64], a TR of 3200 ms, TE of 85 ms and a slice thickness of 2.0 mm. B0 and additional b-values of 1000 and 3000 s/mm^2^ will be used in 101 gradient directions. During DWI, participants are presented with a fixation cross. Afterwards, a scan will follow in the opposite phase encoding direction to facilitate corrections during postprocessing.

Multi-PLD pCASL will provide information on differences in brain perfusion. Max. labeling duration is 1800 ms and fifteen PLDs will be employed: 900 ms, 1200 ms, 1500 ms, 1700 ms, 1800 ms, 1900 ms, 2000 ms, 2100 ms, 2200 ms, 2300 ms, 2500 ms, 2800 ms, 3100 ms, 3400 ms, 3700 ms. These timepoints will allow for accurate perfusion mapping in controls without pain, while also providing a margin for possibly delayed responses in participants in pain [65]. These scans have a TR of 4400.0 ms, TE of 21.70 ms and a slice thickness of 4.0 mm. Participants are once again presented with a fixation cross during the pCASL scans. The total MRI scan will have a duration of approximately 55 minutes.

### 2.9. Pain recovery or persistence

The General Perceived Recovery (GPR) scale will be used to assess recovery or persistence of LBP. Patients will be dichotomized as (1) either being recovered at six-month follow-up based on pain intensity <2/10 indicated on a VAS, no pain-related disability indicated by a PDI score <14/70, and recovery indicated on a 11-point VAS whereby patients rate perceived recovery from −5 (vastly worse) to +5 (greatly improved), with zero corresponding to ‘no change’, rated on average over the past month, or (2) being non-recovered or persisting when not fulfilling these criteria, and since the duration exceeded six months, their MSK pain is considered chronic [24].

### 2.10. Statistical analysis

A sample size calculation was performed using G*Power 3.1. A medium effect size was taken as a conservative omnibus estimation in relation to the stress assessments, based on previous studies. Large to very large effects have been observed for chronic stress [66] and basal cortisol levels [53]. Furthermore, acute stress inductions often failed to yield reliable cortisol responses, but medium to large effects have been observed for stress reactivity associations with self-reported pain responses [22].

Based on a one-way omnibus ANOVA (similar, but slightly more conservative than mixed linear models) with four groups, α of.05, desired power of.80, and a medium effect size of f = .30, 128 participants in total (n = 32 across four groups) are required. Accounting for corrupt or missing data, a sample of n = 35/group will be recruited. Additional sensitivity analyses were executed to calculate the observable effect size based on this sample in the longitudinal arm of the study (parameters: α = .05, power = .80, two groups, two measurements, correlation among repeated measures.2−.6; total sample n = 56 to account for 20% dropout/loss to follow-up). The calculation indicated that small to medium effects would be detectable (f = .17−.24) for within-between interactions in a repeated measures ANOVA.

The different outcome measures will be compared between participant groups and between different time points, using appropriate statistical tests (e.g., linear mixed model, ANOVA). Correlation analysis will be used to examine pain-stress interactions. For the longitudinal arm, analyses will focus on changes over time (i.e., baseline versus six-month follow-up), group comparisons based on pain status (i.e., recovered vs persisting) at follow-up, and interactions between changes over time in stress system functioning and the pain groups. At its core will be a linear mixed model analysis with ‘time’ as the within-subjects factor and ‘pain status group’ as the between-subjects factor. Post-hoc comparisons and adjustments for multiple comparisons will be made using Bonferroni-correction, unless more suitable post-hoc tests are identified. For all statistical tests, statistical significance will be accepted at an α of.05.

### 2.11. General hypotheses

It is hypothesized that the pain groups that vary in pain extent and duration will show distinct profiles of stress system functioning. We expect that individuals with chronic widespread pain (fibromyalgia) will exhibit the most pronounced dysregulation of the stress system, characterized by lower basal cortisol levels due to chronic HPA axis exhaustion, blunted acute stress reactivity, prolonged stress recovery, increased ANS dysfunction with elevated sympathetic tone and reduced parasympathetic reactivation, and greater alterations in cortico-limbic circuitry, which will be associated with amplified pain sensitivity and impaired pain inhibition. Individuals with chronic localized LBP are expected to exhibit moderately dysregulated stress responses, with a mix of heightened and blunted HPA axis reactivity depending on pain extent and duration, increased basal stress levels as reflected in higher cortisol concentrations compared to pain-free controls, mild-to-moderate disruptions in ANS regulation, potentially less severe than in fibromyalgia and associated with impaired pain modulation. Individuals with subacute localized LBP are expected to display a more intact but still altered stress response compared to chronic pain groups, with heightened stress reactivity but preserved recovery, and increased risk for pain chronification (i.e., pain persistence at the six month follow-up) if they exhibit persistent stress exposure, higher basal stress levels, blunted stress reactivity, slower recovery, stress-induced hyperalgesia, and/or alterations in cortico-limbic circuitry. Pain-free individuals are hypothesized to exhibit a balanced stress response, with adequate stress reactivity and efficient recovery from acute stress induction, and the effective endogenous pain modulation compared to all pain groups.

## 3. Discussion

This study will be the first to comprehensively map the functioning of the stress systems (i.e., ANS and HPA axis) and investigate its relationship with pain sensitivity and modulation across groups categorized by pain duration and extent, as well as its role in pain recovery and chronification.

MSK pain, especially low back pain, is the primary cause of disability and affects more than half a billion people worldwide [67]. Given the limited understanding of the role of stress in the extent of pain symptomatology, its contribution to the risk of pain chronicity, and mechanisms underlying the interaction between stress and pain—coupled with the significant heterogeneity in the existing literature—this study aims to provide novel and critical insights into the psychosocial, (psycho)physiological and neural mechanisms underlying the stress-pain relationship. This is an indispensable prerequisite before we can take the next steps to examine and develop well-targeted and successful prevention strategies and treatments for acute and chronic MSK pain.

## Supporting information

S1 Table Overview of measurements. (DOCX)

S1 Data STRAIN_study_protocol_approved. (PDF)

S2 Data STRAIN_Overview_Measurements. (DOCX)
