# Correction: A comprehensive mapping of stress system interactions with pain and their contribution to chronification of musculoskeletal pain: Protocol of the STRAIN study

**DOI:** 10.1371/journal.pone.0331107

**Published:** 2025-08-26

**Authors:** Matthijs Moerkerke, Joren Vyverman, Robrecht De Baere, Patricia Clement, Tom Smeets, Iris Coppieters, Inge Timmers, Jessica Van Oosterwijck

The authors, Jessica Van Oosterwijck and Inge Timmers, should be noted as shared co-corresponding authors on this work. Jessica Van Oosterwijck’s email address is: Jessica.VanOosterwijck@UGent.be and Inge Timmers’s email address is: Timmers@tilburguniversity.edu.
