# Supplementary material for: A comprehensive mapping of stress system interactions with pain and their contribution to chronification of musculoskeletal pain: Protocol of the STRAIN study
Source: PLoS One. 2025 Jun 24;20(6):e0324089. doi: 10.1371/journal.pone.0324089 (PMC12186961; doi:10.1371/journal.pone.0324089)
Supplement: S1 Table — (DOCX) [file pone.0324089.s001.docx]

| MeASUREMENT | Screening (T0) | 10 daYS BEFORE (=enrollement) cross-sectionAL STUDY VISIT (T1) | Cross-sectionAL STUDY VISIT (T2) | DaY 1 cross-sectionAL sALIVA SAMPLE AT HOME (T3) | DaY 2 cross-sectionAL SALIVA SAMPLE AT HOME (T4) | DaY 3 cross-sectionAL SALIVA SAMPLE AT HOME (T5) | 10 daYS BEFORE (=enrolement)  longitudinaL STUDY VISIT (T6) | LongitudinAL STUDY VISIt (T7) | DaY 1 longitudinaL sALIVA SAMPLE AT HOME (T8) | DaY 2 longitudinaL sALIVA SAMPLE AT HOME (T9) | DaY 3 longitudinaL SALIVA SAMPLE AT HOME (T10) |
| --- | --- | --- | --- | --- | --- | --- | --- | --- | --- | --- | --- |
| SURVEYS/QUESTIONNAIRES | | | | | | | | | | | |
| ICF1 | ✔️ |  |  |  |  |  |  |  |  |  |  |
| STRAIN Screening GENERAL | ✔️ |  |  |  |  |  |  |  |  |  |  |
| STRAIN Screening LBP | ✔️ |  |  |  |  |  |  |  |  |  |  |
| DatE STUDY VISIT INCLUSION | ✔️ |  |  |  |  |  |  |  |  |  |  |
| MINI Interview | ✔️ |  |  |  |  |  |  |  |  |  |  |
| ICF2 |  | ✔️ |  |  |  |  |  |  |  |  |  |
| GPR Scale |  |  |  |  |  |  | ✔️ |  |  |  |  |
| STRAIN DEMOGRAPHIC QUESTIONNAIRE |  | ✔️ |  |  |  |  |  |  |  |  |  |
| STRAIN PAINSPECIFIC QUESTIONNAIRE |  | ✔️ |  |  |  |  | ✔️ |  |  |  |  |
| STRAIN LBP QUESTIONNAIRE |  | ✔️ |  |  |  |  | ✔️ |  |  |  |  |
| STRAIN FM QUESTIONNAIRE |  | ✔️ |  |  |  |  |  |  |  |  |  |
| HADS |  | ✔️ |  |  |  |  | ✔️ |  |  |  |  |
| BRS |  | ✔️ |  |  |  |  | ✔️ |  |  |  |  |
| IUS-12 |  | ✔️ |  |  |  |  | ✔️ |  |  |  |  |
| SMM |  | ✔️ |  |  |  |  | ✔️ |  |  |  |  |
| PSS |  | ✔️ |  |  |  |  | ✔️ |  |  |  |  |
| PASS-20 |  | ✔️ |  |  |  |  | ✔️ |  |  |  |  |
| PCS |  | ✔️ |  |  |  |  | ✔️ |  |  |  |  |
| PVAQ |  | ✔️ |  |  |  |  | ✔️ |  |  |  |  |
| PSQI - adapted |  | ✔️ |  |  |  |  | ✔️ |  |  |  |  |
| PDI | ✔️ | ✔️ |  |  |  |  |  |  |  |  |  |
| CTQ |  | ✔️ |  |  |  |  |  |  |  |  |  |
| GEN-P |  | ✔️ |  |  |  |  | ✔️ |  |  |  |  |
| Strain DaILY REPORT |  |  | ✔️ |  |  |  |  | ✔️ |  |  |  |
| STRAIN QUESTIONNAIRE CURRENT LBP |  |  | ✔️ |  |  |  |  | ✔️ |  |  |  |
| STRAIN QUESTIONNAIRE CURRENT FM |  |  | ✔️ |  |  |  |  |  |  |  |  |
| PANAS BASELINE |  |  | ✔️ |  |  |  |  | ✔️ |  |  |  |
| STRAIN VAS PRE |  |  | ✔️ |  |  |  |  | ✔️ |  |  |  |
| PANAS PRE |  |  | ✔️ |  |  |  |  | ✔️ |  |  |  |
| PASA |  |  | ✔️ |  |  |  |  | ✔️ |  |  |  |
| STRAIN VAS POST |  |  | ✔️ |  |  |  |  | ✔️ |  |  |  |
| PANAS POST |  |  | ✔️ |  |  |  |  | ✔️ |  |  |  |
| PHYSIOLOGICAL stress MEASUREMENTS | | | | | | | | | | | |
| ANS measures |  |  | ✔️ |  |  |  |  | ✔️ |  |  |  |
| Biochemical MEASUREMENTS | | | | | | | | | | | |
| HAIR SAMPLE |  |  | ✔️ |  |  |  |  | ✔️ |  |  |  |
| SALIVA SAMPLE |  |  | ✔️ | ✔️ | ✔️ | ✔️ |  | ✔️ | ✔️ | ✔️ | ✔️ |
| stress induction (tsst) |  |  | ✔️ |  |  |  |  | ✔️ |  |  |  |
| Experimental pain measures (qst) |  |  | **✔️** |  |  |  |  | **✔️** |  |  |  |
| neural measures (mri) |  |  | ✔️ |  |  |  |  | ✔️ |  |  |  |
