# Supplementary material for: A comprehensive mapping of stress system interactions with pain and their contribution to chronification of musculoskeletal pain: Protocol of the STRAIN study
Source: PLoS One. 2025 Jun 24;20(6):e0324089. doi: 10.1371/journal.pone.0324089 (PMC12186961; doi:10.1371/journal.pone.0324089)
Supplement: S1 Data — (PDF) [file pone.0324089.s002.pdf]

A comprehensive mapping of stress system interactions with pain and their contribution to chronification of musculoskeletal pain: STRAIN study

SHORT TITLE: The Role of Stress in Pain (STRAIN)

Principal Investigator (PI): Prof. Dr. Jessica Van Oosterwijck
Co-Investigators:
Prof. Dr. Iris Coppieters
Prof. Dr. Inge Timmers
Dr. Matthijs Moerkerke
Drs. Joren Vyverman
Drs. Robrecht De Baere

BACKGROUND

Musculoskeletal (MSK) pain is a major global cause of disability, with a significant personal and socioeconomic impact [1]. To enhance care quality and reduce chronic pain, a better understanding of factors contributing to the development and persistence of chronic MSK pain is urgently needed. One such crucial factor is stress—not only elevated stress levels but also the functioning of stress systems and their interactions with pain processing seem to play an important role. However, existing literature shows conflicting evidence regarding the precise role of stress in pain. Stress has been found to result in both heightened and diminished sensitivity to pain [2, 3].

Interactions between stress parameters of the autonomic nervous system (ANS) (e.g., heart rate variability, respiration rate) and the hypothalamic-pituitary-adrenal (HPA) axis (e.g., cortisol, the "stress hormone") with pain processing parameters have been demonstrated [3]. However, the essence of these relationships has not been extensively mapped. It remains unclear how these stress systems and their parameters behave in different situations: at baseline (i.e., resting state), during stress (i.e., reactivity), and post-stress recovery.

Chronic or repeated stress, which is common in individuals with MSK pain, can strain the body's stress systems (ANS and HPA axis), potentially resulting in hypo- or hyperactivity of these systems [4]. Observations of basal cortisol levels suggest that stress responses vary based on the spread of pain complaints—localized (e.g., low back pain [3]) versus widespread (e.g., fibromyalgia [5]). However, this phenomenon has not been thoroughly investigated across all stress parameters. Additionally, differences in HPA axis responses have been observed between subacute and chronic MSK low back pain patients [6], suggesting that stress system functioning and resilience may play a role in the transition from subacute to chronic pain.

This research hypothesizes that the relationship between stress and pain contributes to vulnerability for pain expansion and chronification. This project aims to characterize stress systems, including reactivity and recovery, in MSK pain while accounting for pain duration and spread. It also seeks to determine how stress influences trajectories of MSK pain, including chronification and recovery. The anticipated results will provide critical insights into the role of stress in the extent of pain symptomatology and chronic pain risk, potentially paving the way for personalized, risk-stratified, stress-focused prevention and rehabilitation strategies.

RESEARCH OBJECTIVES AND HYPOTHESES

Primary Objective:

To characterize stress system functioning, including reactivity and recovery, in individuals with MSK pain, considering pain duration and spread. A combination of psychosocial, (psycho)physiological, and brain measurements will be employed to investigate stress systems and their interaction with pain processing.

Hypothesis: Distinct profiles of ANS and HPA axis dysregulation will be observed among MSK pain groups, with greater deviations in groups experiencing longer pain duration and wider pain spread. These profiles are expected to correlate with pain sensitivity and central pain processing functioning.

Secondary Objective:

To evaluate and define the contribution of stress system functioning to MSK pain trajectories, including pain chronification and recovery. This will involve tracking the evolution of pain complaints, pain processing, and stress system functioning over time, with a focus on differentiating between individuals who recover and those who develop chronic pain.

Hypothesis: Reduced stress reactivity, slower recovery, and elevated chronic stress levels will be associated with pain chronification. Individuals with increased baseline stress levels, altered stress-induced pain sensitivity, and central pain processing abnormalities during the subacute phase are hypothesized to have a higher risk of pain chronification.

PARTICIPANTS

Individuals with MSK pain will be recruited and divided into 3 groups based on pain duration and extent (n=35/group): 1) a group with subacute localized (back) pain, 2) a group with chronic localized (back) pain, and 3) a group with chronic pain throughout the body. Two populations of pain patients will be recruited: i) low back pain (LBP) as localized pain and ii) fibromyalgia (FM) as widespread pain. Additionally, a pain-free control group (n=35) will be recruited. Each group consists of 35 individuals as calculated in an a priori power calculation [3, 7, 8]. Important criteria for the subacute back pain group is pain with an onset <3 months ago and without a clear cause. For the chronic back pain group, this is pain that has been present for more than 6 months [9]. The pain must be of such severity that it impacts daily life, which is assessed using the Pain Disability Index (PDI) and a pain intensity reported of ≥2/10 on a Visual Analog Scale (VAS). Patients with fibromyalgia must provide proof of diagnosis. The pain-free control group must have no history of chronic pain or have sought treatment for a pain complaint <6 months. Participants must be Dutch-speaking and between 18 and 45 years old with a BMI between 18.5 and 35 kg/m2, and must meet specific conditions (such as no contraindication for an MRI, no history of spinal trauma, surgeries or deformities, no current or history of severe psychiatric, neurological, hormonal or cardiovascular conditions, etc.).

To find participants, a recruitment flyer (see appendix) will be distributed both online via social media (e.g., Facebook, Instagram, LinkedIn, X) and physically by posting/placing the printed version in public places (e.g., library). Interested participants will scan the QR code/send an email to STRAIN@UGent.be, after which they can read the online short version of the ICF and give consent to complete the screening questionnaire(s) (redirected to REDCap) (duration ±15 min). The screening consists of a general section with self-constructed questions that probe for inclusion and exclusion criteria regarding personal characteristics such as age, BMI, etc., general health, and the presence of pain complaints. To get an idea of psychological health problems (e.g., post-traumatic stress disorder, major depression) that have a significant impact on the stress system (e.g., on cortisol) and to exclude them, the health survey also includes four short questions from the validated Patient Health Questionnaire (PHQ-4) and one question from the Mini International Neuropsychiatric Interview-short version (MINI-s), namely “Do you think you have been traumatized by one or more events where people (almost) died, were seriously injured, or sexually abused?”. The general survey also asks for contact details so that researchers can contact potential participants. If additional information is needed after the questionnaires have been completed, additional questions will be asked. If additional information is needed about potential psychological health problems that have a significant impact on the stress system, the MINI-S (see appendix) will be used as an interview guide. Participants will be informed by email by the researchers about whether they meet the inclusion criteria and are eligible for study participation. If the participant meets the inclusion criteria, the email will contain a personal link to the detailed long ICF and consent form for the study. If one then decides to participate in the study, the participant must confirm participation in the study on the online consent form. The participant will then receive an email to schedule the study participation.

TEST PROCEDURE

The test procedure consists of four parts: filling out online questionnaires, undergoing (psycho)physiological measurements at UZ Gent/UGent, undergoing MRI scans at UZ Gent/UGent, and collecting saliva samples at the participant's home. The test procedure is identical for each participant, except for some questionnaires that are only completed by participants with pain complaints to characterize these pain complaints. More specifically, participants in the back pain groups and fibromyalgia group will be asked to complete the 'STRAIN Pain-specific Questionnaire' within 10 days before the test moment. Furthermore, participants in the back pain groups will also receive the 'STRAIN Questionnaire LBP' within 10 days before the test moment, and on the day of the experiment, the 'STRAIN Questionnaire Current LBP'. Participants in the fibromyalgia group will receive the 'STRAIN Questionnaire FM' within 10 days before the test moment and complete the 'STRAIN Questionnaire Current Pain Complaints FM' on the day of the experiment. An overview of the test procedure on the test day itself can be found in the appendix (documents).
Upon confirmation of participation, the participant will receive an email notification ten days before the actual test moment with the request to complete the online questionnaires via a personal REDCap link at home (duration ±1 hour). Through this survey, which consists of a mix of self-constructed questions and validated questionnaires, we will examine health-related, pain-related, and stress-related characteristics. The email also contains guidelines for preparing for the test moment that takes place at UZ Gent/UGent. During the test moment, the participant is received in the research lab at the Department of Rehabilitation Sciences at UGent. First, a survey is completed to check compliance with the preparatory guidelines, possible confounding factors, and pain characteristics (e.g., current pain intensity) at the moment itself (duration ±15 min). Then, height and weight are measured.

To assess the functioning of the HPA axis and the chronic stress response, a hair sample is taken by cutting some hair strands (±0.5cm2) from the back of the head [10]. Then, to assess the functioning of the acute stress response of the HPA axis, saliva samples are collected [11]. For this, the participant is asked to take the swab out of the tube and hold it in the mouth for two minutes without chewing on it. The saliva collection is repeated at specific times throughout the study, specifically saliva is collected: 1) at baseline; 2) just before a cognitive acute stress induction task (Trier Social Stress Test); 3) just after the cognitive acute stress induction task; 4) 15 minutes after the cognitive acute stress induction task; 5) 45 minutes after the cognitive acute stress induction task just before going to the MRI scanner.

After the baseline saliva collection, sensors are attached to the skin of the chest and hand for (psycho)physiological measurements. Electrocardiography (ECG) is measured via sensors on the chest. Sensors on the hand are used to determine skin temperature (ST) and skin conductance (SC). Respiratory rate (RR) is recorded using sensors on the chest. These measurements are performed at baseline during a 5-minute rest period and are also captured during and following the evaluation of pain sensitivity, the acute stress induction task, and the MRI scans. Systolic and diastolic blood pressure (BP) are measured discontinuously at the start and end of each measurement period using an electronic blood pressure monitor with an arm cuff. These measurements are performed with the same equipment and in the same manner as in one of our previous studies [12]. After measuring the autonomic parameters at baseline, measurements are performed to evaluate pain sensitivity and pain processing. The pressure pain threshold is determined with a digital algometer at the lower back, leg, and arm [13-15]. The thermal pain and tolerance threshold is assessed using a thermode that heats up until the participant indicates the temperature as painful or no longer tolerable. Furthermore, using the thermode, the sensitivity to one (painful) heat stimulus that is maintained for a longer time (2 minutes) is assessed at the arm, based on scores indicated by the participant [16], and for two (painful) heat stimuli given simultaneously at both arms. This evaluation provides information about pain sensitivity and the functioning of pain-modulating mechanisms [17].

After the evaluation of pressure and heat sensitivity and pain processing, a short survey is conducted to map out the current emotions, stress, and pain. Acute stress is then induced using the widely used and well-validated Trier Social Stress Test (TSST) [18]. The TSST consists of a 5-minute presentation and a short preparation for it (3 minutes), and a 5-minute cognitive arithmetic task. These tasks are performed in front of a "jury" (two people, e.g., researchers of the project) who remain neutral and expressionless during the execution. The participant is informed that the task will be recorded, but the camera will only be focused on the participant to induce stress (no actual recording is made). After the test, the participant is surveyed again to examine the effects of the test on self-reported emotions, stress, and pain. During and after the test, (psycho)physiological autonomic measurements are performed to examine the effects of the test (i.e., reactivity and recovery of the autonomic nervous system). Subsequently,

(psycho)physiological pain measurements are performed again to examine the effects of the test on pain sensitivity and pain processing. During the period after the TSST, saliva samples are collected as previously mentioned to examine the effects of the test on the acute stress response of the HPA axis. The participant is then debriefed, and the ultimate goal of this task is explained.

For the final measurement of the test session, the participant is guided to the Ghent Institute of Metabolic Imaging (GIFMI) (UGent, UZ Gent campus) where they will undergo an evaluation of brain structure and function via MRI. First, an MRI safety checklist (see appendices) will be used to check again for contraindications for scanning (e.g., presence of pacemakers, vascular clips, etc.). The participant then undergoes MRI scans (duration ±1 hour) at rest and during pain stimulation, where MRI-compatible thermodes are used to apply heat stimuli of different temperatures and durations to the arm to evaluate brain activity. Continuous RR, SC, and ECG measurements are taken during the scanning using MRI-compatible sensors. The visit concludes with instructions given to the participant for collecting saliva samples at home, and arrangements are made for the collection of the samples.
During the 3 days following the test session, the participant will collect saliva samples at 5 time points throughout the day. The participant receives an instruction booklet (see appendix) for this. The samples are stored by the participant in the freezer until the researcher collects them.
Participants in the low back pain groups will be re-evaluated with the same measurements after 6 months and divided into 1) recovered or 2) not recovered based on the reported complaints and their impact on daily life [19].
The saliva samples collected during the test session are temporarily stored in the freezer for research at the Department of Rehabilitation Sciences, after which they are sent to the UZ Gent biobank for splitting of the saliva samples. Part of the sample is stored in the biobank, and part is sent to Dresden LabService for analysis. Cortisol levels, oxytocin levels, and alpha-amylase levels will be determined in the saliva samples, and cortisol levels will be determined in the hair samples. After the analyses, Dresden LabService will destroy the samples.
The main institute is UGent, led by Prof. Dr. Jessica Van Oosterwijck, where data collection will also take place. Funding for this project was obtained through the FWO, with UGent being the main Flemish institute and Prof. Dr. Jessica Van Oosterwijck as supervisor/spokesman-PI, and Vrije Universiteit Brussel as the additional Flemish host institution with Prof. Dr. Iris Coppieters as supervisor. An additional non-Flemish partner is Tilburg University, where Prof. Dr. Inge Timmers is co-supervisor and also a guest professor at UGent. This research involves 2 predoctoral researchers appointed with the FWO funding obtained for this project: Robrecht de Baere, joint-PhD student at Vrije Universiteit Brussel and UGent; and Joren Vyverman, joint-PhD student at UGent and Tilburg University. For both PhD students, Prof. Dr. Jessica Van Oosterwijck is the administrative promoter on the UGent side. For Joren Vyverman, Prof. Dr. Inge Timmers is the promoter on the Tilburg side. For Robrecht de Baere, the administrative promoter (Prof. Dr. Iris Coppieters) is at Vrije Universiteit Brussel. Data collection takes place at UGent.

Key references: [1] Vos et al. The Lancet. 2012; [2] Crettaz et al. PloS One. 2013; [3] Vachon-Presseau et al. J Neurosci. 2013; [4] Woda et al. Psychoneuroendocrinology. 2016; [5] Freitas et al. J Back Musculoskelet Rehabil. 2012; [6] Timmers et al. Neurosci Biobehav Rev. 2019; [7] Van Uum et al. Stress. 2008; [8] Vachon-Presseau et al. Brain. 2013; [9] Merskey et al. IASP press. 1994; [10] Greff et al. Clin Biochem. 2019; [11] Timmers et al. European Journal of Pain. 2018; [12] Van Oosterwijck et al. Pain Physician. 2017; [13] Graven-Nielsen et al. Arthritis Rheum. 2012; [14] Giesbrecht et al. Phys Ther. 2005; [15] Meeus et al. J Rehabil Med. 2010; [16] De Vita et al. Pain Medicine. 2022; [17] Geva & Defrin. The Journal of Pain. 2018; [18] Allen et al. Neurobiology of Stress. 2017; [19] Kamper et al. Eur Spine J. 201
